# Locust bean gum adsorption onto softwood kraft pulp fibres: isotherms, kinetics and paper strength

**DOI:** 10.1007/s10570-021-04133-w

**Published:** 2021-10-01

**Authors:** Jingqian Chen, Rodger P. Beatson, Heather L. Trajano

**Affiliations:** 1 Department of Chemical and Biological Engineering, University of British Columbia, 2360 East Mall, Vancouver, BC V6T 1Z3 Canada; 2 BioProducts Institute, University of British Columbia, Vancouver, BC V6T 1Z3 Canada; 3 Chemical and Environmental Technology, British Columbia Institute of Technology, 3700 Willingdon Avenue, Burnaby, BC V5G 3H2 Canada

**Keywords:** Northern bleached softwood kraft pulp, Paper strength additive, Hemicellulose, Locust bean gum, Adsorption isotherms, Adsorption kinetics

## Abstract

The adsorption of locust bean gum (LBG) onto Northern Bleached Softwood Kraft (NBSK) pulp improved paper tensile and burst strength and lowered refining energy by strengthening inter-fibre bonding. Adsorption kinetics and isotherms were investigated to develop a fundamental understanding of the adsorption mechanism. The adsorption rate followed pseudo-second-order kinetics and the activation energy was 99.34 kJ·mol^−1^, suggesting chemisorption. The adsorption rate constant increased rapidly with temperature from 25 to 45 °C (*k* = 1.93 to 24.03 g·mg^−1^·min^−1^), but the amount adsorbed at equilibrium decreased (*q*_*e*_ = 1.91 to 0.48 mg·g^−1^ o.d. fibre). LBG adsorption to NBSK at 25 °C was consistent with the Langmuir adsorption model for LBG < 2.1 wt% of o.d. fibre, suggesting reversible, homogenous adsorption to a finite number of sites on the fibre surface. Refining to 3000 rev increased the heterogeneity of the NBSK pulp surface leading to multi-layer Freundlich adsorption with adsorption constant *n* = 5.00, and the equilibrium constant *K*_*f*_ = 2.57 mg·g^−1^·(mg·L^−1^)^−1/n^ at 25 °C. Favorable adsorption conditions for negatively charged LBG were identified: 25 °C for 10 min, low dosage level (< 2 wt%), lightly refined (< 3000 rev) NBSK pulp at low fibre consistency (< 0.5 wt%), high agitation rate (> 150 r.p.m.), acidic or neutral conditions (pH 2–7) without salt addition.

**Graphic abstract:**

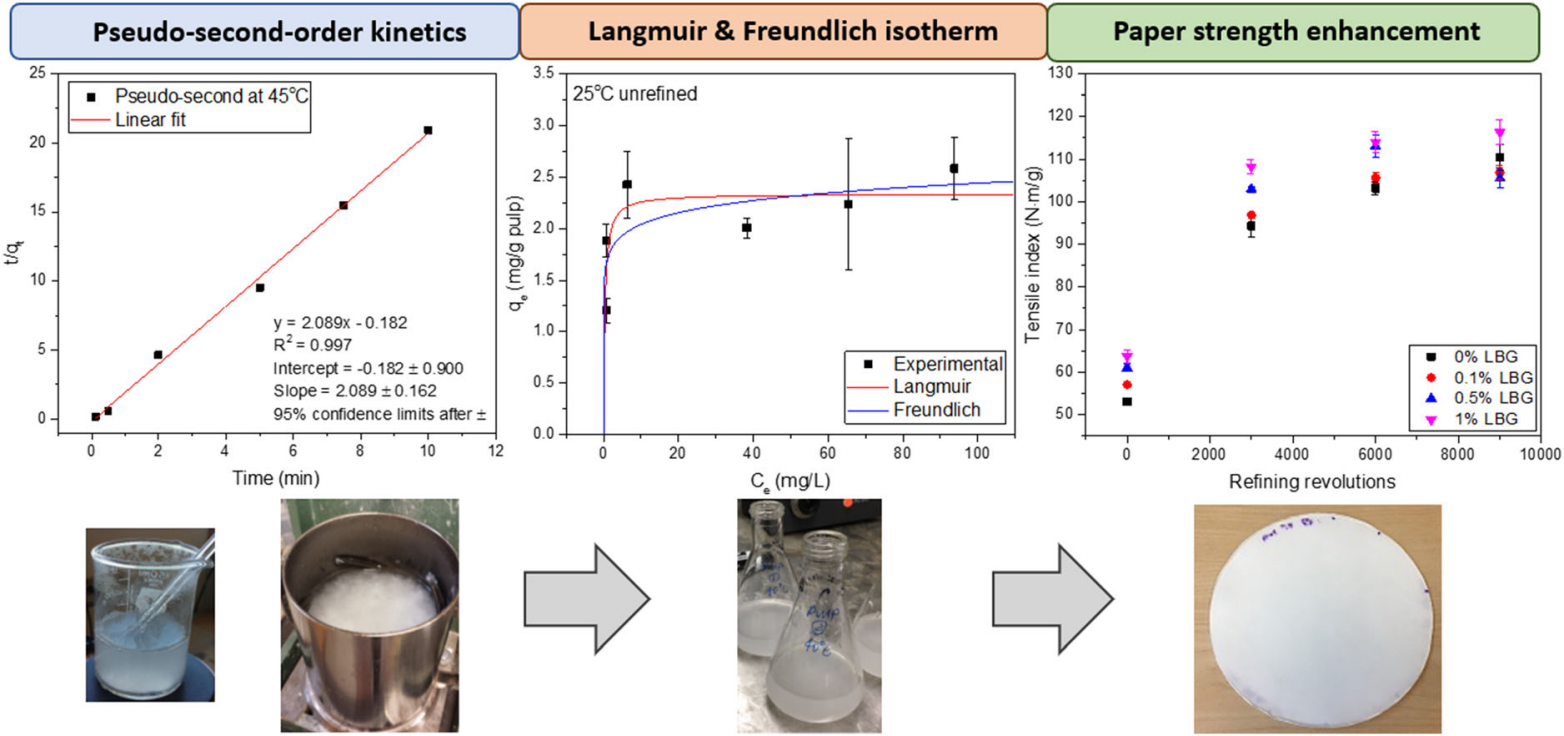

**Supplementary Information:**

The online version contains supplementary material available at 10.1007/s10570-021-04133-w.

## Introduction

Paper strength, essential to many applications, relies on the number of interfibre bonds, the strength of the bonds, fibre properties (e.g. fibre strength, length, and coarseness), and the distribution of fibres and bonds (sheet formation) (Niskanen 1998; Lindström et al. 2005; Leech 1953, 1954). Paper strength can be modified through mechanical refining and the use of additives such as polysaccharides. Softwood grown in the Northern hemisphere (e.g. British Columbia, Canada) produces high-strength bleached kraft pulp; finding ways to further enhance strength properties while reducing refining energy is a key objective for Northern Bleached Softwood Kraft (NBSK) producers.

Increasing paper strength through refining consumes large amounts of mechanical energy. Strength improvement through application of additives after refining reduces the energy required to achieve target paper strength (Bhaduri et al. 1995; Silva et al. 2010). The earliest paper strength additives were polysaccharides with structural affinity for cellulose such as locust bean gum (LBG) and guar gum (Leech 1953; Lindström et al. 2005; Most 1957; Russo 1959; Swanson 1950). Leech (1954) concluded that 0.5 wt% LBG dosage doubled the bonding strength of paper. Swanson (1950) reported that tensile strength increased by 33% following sorption of 2 wt% LBG to coniferous sulphite pulp. Burst strength increased 32% with 0.5 wt% LBG in beaten pulp; this corresponded to a 70% reduction in beating time (Swanson 1950). Paper strength enhancement by hemicellulose adsorption results from an increased number of bonds plus increased bonded area and bond strength (Leech 1954). The primary bonding between fibres and between hemicellulose and fibres is hydrogen bonding (Niskanen 1998; Leech 1954; Hannuksela et al. 2002).

Many factors have been shown to influence adsorption of hemicellulose onto pulp fibre: adsorption conditions (Russo 1959; Gruenhut 1953; Leech 1953; Most 1957; Swanson et al. 1949), fibre properties (Zakrajšek et al. 2009), and hemicellulose properties (Hannuksela et al. 2002, 2004; Lindqvist et al. 2013). Given the multitude of factors and interactions, there are many contradictory reports regarding the effects of changing variables on adsorption results.

Adsorption conditions include temperature, time, hemicellulose dosage, pH, salt addition, fibre consistency and agitation rate. Temperature and time are the most commonly examined factors for hemicellulose adsorption, but the reported effects vary greatly. Adsorption of partially methylated LBG on bleached sulfite pulp increased with temperature from 5 to 61 °C (Russo 1959). However, Gruenhut (1953) concluded that LBG adsorption to kraft fibre increased with decreasing temperature; maximum adsorption was observed at 4.2 °C. Most (1957) and Leech (1953) reported a greater amount of hemicellulose was retained by pulp fibre with increasing time, and further concluded that adsorption equilibrium was not obtained even after 10 days. However, Swanson et al. (1949) obtained 76–96% LBG adsorption to bleached sulfite pulp and reported that equilibrium was reached within 30 min.

Salts, process chemicals and pH also strongly influence the process (Hedborg and Lindström 1993; Shirazi et al. 2003; van de Steeg 1989; van de Steeg et al. 1993a, b; Zakrajšek et al. 2009) since adsorption occurs by electrostatic interaction of polyelectrolytes (polymers with electrolyte groups) with negatively-charged cellulose fibres (Niskanen 1998; van de Ven 2000; Sjostrom 1989). Cellulose fibres are negatively charged due to carboxyl groups and hydroxyl groups. Addition of salts decreases the attractive electrostatic forces between cationic starch and cellulose fibre thus adsorption decreases (van de Steeg 1989; van de Steeg et al. 1993a, b; Hedborg and Lindström 1993). When pH increases, carboxyl groups deprotonate and generate more negative charge on fibre surface (Hedborg and Lindström 1993). As a result, adsorption of cationic polymers increases with rising pH (Shirazi et al. 2003; van de Steeg 1992; van de Steeg et al. 1993a, b). However, for polymers with negative charge, low pH facilitates adsorption by converting carboxyl groups to their undissociated state (Scallan 1983). High pH leads to a high electrostatic repulsion between fibres and negatively charged polymer, thus reducing the adsorption. Gruenhut (1953) concluded that LBG adsorption to kraft pulp fibre was higher at pH 4 than at pH 6.5. Keen and Opie (1957) found that maximum guar gum adsorption to bleached kraft pulp was obtained at pH 6.7 and minimum adsorption occurred at pH 11.5. In contrast, Most (1957) found hemicellulose from slash pine adsorbed more to bleached sulfite pulp at pH 10 than at pH 4.5. Finally, Hannuksela et al. (2002) reported that adsorption of guar gum on bleached kraft pulp was independent of refining severity, pH, temperature and salt concentration. de Jong and van de Velde (2007) determined that the charge density, defined as mol negative charge/mol of monosaccharide, of native LBG was less than 0.3. Thus, LBG is a weakly, negatively charged polymer, and salts might have a relatively low impact on adsorption. The results of the limited number of studies on the influence of ionic strength and pH on hemicellulose adsorption to cellulose are contradictory.

Mass transfer, another important adsorption condition, is influenced by agitation rate and fibre consistency. Turbulence, created by strong agitation, reduces mass transfer resistance by disrupting the boundary layer at the interface of the fibre and bulk solution (Russo 1959; Zakrajšek et al. 2009). Fibre consistency negatively correlates to extent of adsorption. Zakrajšek et al. (2009) and Most (1957) showed low fibre consistency increased adsorption of starch and hemicellulose to pulp fibres due to high concentration gradient and greater fibre surface availability.

Fibre properties such as surface area and fines content change the availability of adsorption sites (Zakrajšek et al. 2009). Several scholars attributed the increase of adsorption as a function of refining due to fibrillation, generation of fines, increased surface area and total pore volume (Zakrajšek et al. 2009; Russo 1959; Keen and Opie 1957; Hannuksela et al. 2002).

To elucidate the contradictory effects of factors, fundamental analysis including adsorption isotherms and kinetics are needed. Adsorption isotherms describe adsorption of a substance to a solid surface from an aqueous phase under isothermal conditions (Foo and Hameed 2010). Langmuir isotherms and Freundlich isotherms are commonly used to describe dye or chemical adsorption to cellulosic fibres (Langmuir 1916; Li et al. 2018; Roy et al. 2013; Urruzola et al. 2013; Vučurović et al. 2012; Zakrajšek et al. 2009).

Adsorption kinetics describe the variation of amount adsorbed with time and can guide how to most effectively apply additives during papermaking (Zakrajšek et al. 2009). Adsorption rates of polymers are related to the collision rate. For small particles, collision rate is dependent on Brownian motion, while for large particles or systems with flow motion, the rate is dependent on flow conditions (van de Ven 1994). Pseudo-first-order and pseudo-second-order models are commonly used to describe the kinetics of adsorption of dyes or chemicals to pulp fibres. They were proposed (Lagergren 1898; Ho 1995, 2006; Ho et al. 1996; Blanchard et al. 1984; Ho and McKay 1998, 2000) and applied in many cellulose/fibre adsorption studies (Li et al. 2018; Roy et al. 2013; Vučurović et al. 2012; Pan et al. 2016). However, reports on application to polysaccharides adsorption to pulp fibre are rare.

Currently, starch is the most widely used strength additive thus its adsorption is well studied (Hedborg and Lindström 1993; Wågberg and Bjorklund 1993; van de Steeg 1989; van de Steeg et al. 1993a, b; van de Steeg 1992; Shirazi et al. 2003; Zakrajšek et al. 2009). However, hemicelluloses recovered from wood, such as O-acetyl-galactoglucomannans (GGM), could also be used as strength additives. These could be isolated from process streams in the pulp and paper mills as part of an integrated biorefinery. There is limited fundamental understanding of application of hemicellulose polysaccharides such as GGM, or the closely-related LBG. GGM recovered from pulp mill wastes has high polydispersity (Chen et al. 2020), are contaminated with other biomass components (e.g. extractives) and thus are is not well-suited to studies investigating fundamental adsorption mechanisms. In contrast, LBG is a well-defined, commercially available galactomannan-type polysaccharide: a backbone of (1–4)-β-D-mannopyranosyl units with side chains of (1–6)-α-D-galactopyranosyl units having a 1:4 ratio of galactose to mannose (BeMiller and Whistler 2012; Roller and Jones 1996). The composition, molecular structure and charge density of starch and LBG differ considerably thus past starch research cannot be transferred to LBG adsorption.

The goals of this work are to identify the favorable adsorption conditions for LBG on NBSK pulp, understand the underlying mechanisms, and the resulting effects on paper properties. LBG adsorption is analyzed using pseudo-first-order and pseudo-second-order kinetics with respect to LBG concentration. The adsorption isotherms are analyzed using both the Langmuir model and the Freundlich model. The effects of temperature, refining, sodium chloride addition, and pH on LBG adsorption are investigated. Changes in paper strength due to LBG adsorption to unrefined and refined NBSK pulp are investigated; refining and LBG dosage are varied.

## Materials and methods

### Materials

Never dried NBSK pulp was supplied by Canfor Pulp Products. The NBSK pulp was washed with deionized water until the UV–vis absorption of filtrate was less than 0.005 abs at 200 nm wavelength before use. LBG with purity greater than 90% was purchased from Sigma Aldrich. Sodium chloride (> 99%), potassium chloride (> 99%), hydrochloric acid (37%), sodium acetate (> 99%), acetic acid (> 99%), sodium bicarbonate (> 99%), sodium carbonate (> 99%), potassium chloride (> 99%) and sodium hydroxide (> 98%) were purchased from Sigma Aldrich. Sodium phosphate monobasic (> 98%) and dibasic (> 99%) were purchased from Fisher Scientific. Sulfuric acid (98 wt%) was purchased from Sigma Aldrich and diluted to desired concentration. The carbohydrates kit (CAR10-1KT) used to calibrate the high-performance liquid chromatography (HPLC) was purchased from Sigma Aldrich and contained mannose, glucose, galactose, xylose and arabinose. The purity of the standards was greater than 98%.

### LBG adsorption

LBG powder was hydrolyzed in deionized water at a concentration of 0.5 wt% at 98–100 °C for 45 min with continuous agitation to produce a solution of polysaccharides with a narrow molar mass distribution. Undissolved gum particles in hydrolyzed LBG stock solution were removed by two rounds of vacuum filtration. The filtrate was further diluted and centrifuged twice at 3500 r.p.m. for 15 min, and the supernatant was recovered for adsorption experiments. The weight-average molar mass of hydrolyzed LBG was measured by a Waters Alliance HPLC coupled with refractive index (RI) detector and Ultrahydrogel 120, 250, and 1000 columns. The calibration standard was a pullulan standard kit (WAT034207). The weight-average molar mass of LBG was 1215 kDa (± 89 kDa standard deviation) in this work.

Adsorption experiments were conducted in capped 150 mL Erlenmeyer flasks. Control flasks containing only LBG or only fibre were run in parallel. The galactomannan detected in the supernatant of the fibre-only control was 0.14 mg·g^−1^ o.d. fibre (standard deviation of 0.05 mg·g^−1^). All conditions were tested as 2–4 replicates. After adsorption, all samples were centrifuged at 3500 r.p.m. for 15 min. The supernatant was preserved for compositional analysis.

The experimental design for LBG adsorption kinetics and isotherm is summarized in Table 1. The kinetics study was conducted by adding 0.12–0.21 wt% LBG relative to o.d. pulp fibre (0.21wt% at 25 °C, 0.14wt% at 35 °C, and 0.12wt% at 45 °C); the fibre consistency of the slurry was 0.5 wt%. Adsorption was conducted at 25 °C, 35 °C, and 45 °C in an incubator shaker with continuous agitation (150 r.p.m.) for 0.5 to 120 min for NBSK unrefined pulp. Adsorption isotherm experiments were conducted by varying LBG dosage from 0.1 to 2.1 wt% of o.d. pulp fibre. The PFI mill (Noram Quality Control & Research Equipment Limited) was used to refine the pulp to 3000 rev to compare with unrefined NBSK pulp. Adsorption was conducted at 25 °C and 35 °C with continuous agitation (150 r.p.m.) for 10 min in the incubator shaker for unrefined NBSK pulp. The isotherm experiments for refined NBSK pulp were conducted by varying LBG dosage from 0.2 to 0.6 wt% of o.d. pulp fibre at 25 °C.

**Table 1:** LBG adsorption kinetics and isotherm experimental design for unrefined and refined NBSK pulp. Factors investigated for LBG adsorption: LBG dosage, temperature, NaCl addition, and pH. Note: standard deviation after ±

Use	Refining (rev)	Dosage relative to o.d. pulp (wt%)	Adsorption temperature (°C)	Adsorption time (min)	NaCl addition (mol·L^−1^)	pH of sample suspension
Kinetics	0	0.12–0.21	25, 35, 45	0.5–120	0	5.33 ± 0.35
Isotherm	0	0.1–2.1	25, 35	10	0	5.43 ± 0.35
Isotherm	3000	0.2–0.6	25	10	0	5.04 ± 0.41
NaCl effect	0	0.2	25	10	0–1	5.08 ± 0.56
pH effect	0	0.2	25	10	0	2–13

Table 1 also summarizes the conditions tested to determine the effect of LBG concentration, temperature, salt addition, and pH on adsorption. All trials were performed for 10 min with continuous agitation of 150 r.p.m. Sodium chloride was varied from 0–1 mol·L^−1^ and pH was varied from 2 to 13 in order to test the full range of conditions previously reported in the literature. The buffer (0.1 M) was prepared with potassium chloride and hydrochloric acid (pH 2), sodium acetate and acetic acid (pH 5), sodium phosphate monobasic and dibasic (pH 7), sodium bicarbonate and sodium carbonate (pH 10), and potassium chloride and sodium hydroxide (pH 13).

### LBG solution compositional analysis

The galactomannans were hydrolyzed from polysaccharides to monosaccharides with sulfuric acid in an autoclave at 121 °C for 1 h according to National Renewable Energy Laboratory Analytical Procedures (Sluiter et al. 2008, 2006; Hames et al. 2008). The galactomannan monomer content in the supernatant was analyzed by Dionex AS50 HPLC (Thermo Scientific) coupled with an ion exchange PA1 column (Dionex), an ED50 electrochemical detector (pulsed amperometric detector) with a gold electrode, and an AS50 autosampler (Dionex). Deionized water was used as eluent with a flow rate of 1 mL·min^−1^. The auxiliary pump added 0.2 M NaOH at 0.5 mL·min^−1^. The samples were filtered through a 0.22 μm nylon syringe filter before injection. The injection volume was 10 μL.

The fraction of LBG adsorbed to the pulp, *f*_*L*_, was determined by the difference of galactomannan content in supernatant, relative to LBG control after adsorption:

1 $$f_{L} = \frac{{C_{0F} + C_{0L} - C_{SL} }}{{C_{0L} }} \times 100\%$$

where *C*_*0F*_ is the galactomannan concentration (mg·L^−1^) detected in supernatant of fibre control flask, *C*_*0L*_ is initial LBG concentration (mg·L^−1^), calculated from LBG control flask and *C*_*SL*_ is LBG concentration in supernatant (mg·L^−1^) after adsorption.

The absolute amount of LBG, *m*_*L*_, adsorbed to the pulp (mg·g^−1^ o.d. fibre) was calculated as:

2 $${\text{m}}_{{\text{L}}} = \frac{{{\text{C}}_{{{\text{0F}}}} + {\text{C}}_{{0{\text{L}}}} - {\text{C}}_{{{\text{SL}}}} }}{{{\text{C}}_{{0{\text{P}}}} }}$$

where *C*_*0P*_ is the initial o.d. pulp fibre concentration (g·L^−1^).

### Adsorption kinetics and isotherms

The Langmuir isotherm model assumes ideal monolayer chemisorption on a smooth surface with a finite number of sites (Langmuir 1916; Laidler 1987; Foo and Hameed 2010). The Langmuir isotherm is derived from the equilibrium adsorption reaction of LBG to substrate NBSK pulp fibre:

3 $${\text{C}}_{{\text{e}}} + {\text{ S}}_{{\text{e}}} \leftrightarrow {\text{ C}}_{{\text{e}}} {\text{S}}_{{\text{e}}}$$

where *C*_*e*_ is the equilibrium concentration of LBG (mg·L^−1^) in the aqueous phase, *S*_*e*_ is the concentration of empty sites at equilibrium (mg·g^−1^ o.d. fibre) on the surface of pulp fibres, and *C*_*e*_*S*_*e*_ is the equilibrium concentration of adsorbed LBG (mg·g^−1^ o.d. fibre) on fibre surface.

When at equilibrium, *k*_*1*_ is the adsorption rate constant (L·g·mg^−2^) and *k*_*-1*_ is the desorption rate constant (g·mg^−1^).

4 $${\text{k}}_{1} [{\text{C}}_{{\text{e}}} \left] {\left[ {{\text{S}}_{{\text{e}}} } \right] = {\text{k}}_{ - 1} } \right[{\text{C}}_{{\text{e}}} {\text{S}}_{{\text{e}}} ]$$

Defining substrate surface coverage as *θ*, then *S*_*e*_ can be expressed as Eq. 6:

5 $${\uptheta } = \frac{{\left[ {{\text{C}}_{{\text{e}}} {\text{S}}_{{\text{e}}} } \right]}}{{\left[ {{\text{S}}_{{\text{e}}} } \right] + \left[ {{\text{C}}_{{\text{e}}} {\text{S}}_{{\text{e}}} } \right]}}$$

6 $$\left[ {{\text{S}}_{{\text{e}}} } \right] = \left( {1 - {\uptheta }} \right)\left( {\left[ {{\text{S}}_{{\text{e}}} } \right] + \left[ {{\text{C}}_{{\text{e}}} {\text{S}}_{{\text{e}}} } \right]} \right)$$

Equations 4, 5 and 6 can be combined to yield Eq. 7:

7 $${\text{k}}_{1} [{\text{C}}_{{\text{e}}} ]\left( {1 - {\uptheta }} \right) = {\text{k}}_{ - 1} {\uptheta }$$

The equilibrium constant *K*_*e*_ (L·mg^−1^) is defined as:

8 $${\text{K}}_{{\text{e}}} = \frac{{{\text{k}}_{1} }}{{{\text{k}}_{ - 1} }} = \frac{{\left[ {{\text{C}}_{{\text{e}}} {\text{S}}_{{\text{e}}} } \right]}}{{\left[ {{\text{C}}_{{\text{e}}} } \right]\left[ {{\text{S}}_{{\text{e}}} } \right]}}$$

Equation 7 and 8 can be combined and further rearranged as Eq. 9. Thus, ${\theta }$ could be solved as Eq. 10:

9 $${\text{K}}_{{\text{e}}} [{\text{C}}_{{\text{e}}} ] = \left( {1 + {\text{K}}_{{\text{e}}} [{\text{C}}_{{\text{e}}} } \right]){\uptheta }$$

10 $${\uptheta } = \frac{{{\text{K}}_{{\text{e}}} [{\text{C}}_{{\text{e }}} ]}}{{1 + {\text{K}}_{{\text{e}}} \left[ {{\text{C}}_{{\text{e}}} } \right]}} = \frac{{{\text{q}}_{{\text{e}}} }}{{{\text{Q}}_{{{\text{max}}}} }}$$

where *q*_*e*_ (mg·g^−1^ o.d. fibre) is equilibrium LBG adsorption capacity and *Q*_*max*_ (mg·g^−1^ o.d. fibre) is maximum adsorption capacity. From Eq. 10, when *C*_*e*_ is large, *θ* is approximately equal to 1, representing full coverage of substrate. In contrast when *C*_*e*_ is small, *θ* approaches zero, suggesting limited adsorption and surface coverage. Nonlinear regression of Eq. 10 was conducted by OriginLab 2016 to determine the *Q*_*max*_ and *K*_*e*_ (Tran et al. 2017).

The rate equation for the Freundlich isotherm (Freundlich 1906; Foo and Hameed 2010) is:

11 $${\text{q}}_{{\text{e}}} = {\text{K}}_{{\text{f}}} {\text{C}}_{{\text{e}}}^{{\frac{1}{{\text{n}}}}}$$

where *K*_*f*_ is the Freundlich equilibrium constant (mg·g^−1^·(mg·L^−1^)^−1/n^), *q*_*e*_ is the concentration of LBG (mg·g^−1^ o.d. fibre) adsorbed at equilibrium state, *n* is the Freundlich constant (dimensionless) related to adsorption intensity and *C*_*e*_ is concentration of LBG (mg·L^−1^) in the aqueous phase (Foo and Hameed 2010; Bergmann and Machado 2015). Nonlinear regression of Eq. 11 was conducted by OriginLab 2016 to determine the *K*_*f*_ and *n* (Tran et al. 2017). The isotherm fitting was assessed by the reduced chi-squared (*χ*^*2*^) and ${{R}}_{{{{adj}}}}^{2}$ as described in Eq. 12 and 13 (Bergmann and Machado 2015).

12 $${\upchi }^{2} = \mathop \sum \limits_{{\text{i}}}^{{\text{N}}} \frac{{\left( {{\text{q}}_{{{\text{i}},{\text{exp}}}} - {\text{q}}_{{{\text{i}},{\text{model}}}} } \right)^{2} }}{{{\text{N}} - {\text{p}}}}$$

13 $$R_{adj}^{2} = 1 - \left( {1 - R^{2} } \right) \cdot \left( {\frac{N - 1}{{N - p - 1}}} \right)$$

where *q*_*i,model*_ is model fitted value of *q*, *q*_*i,exp*_ is experimental value of *q*, *N* is the total number of experiments, and *p* is the number of parameters in the model.

Pseudo-first-order and pseudo-second-order adsorption kinetics (Lagergren 1898; Ho 1995, 2006; Ho et al. 1996; Blanchard et al. 1984; Ho and McKay 2000) are described by Eq. 14 and Eq. 15, respectively:

14 $$\frac{{{\text{dq}}_{{\text{t}}} }}{{{\text{dt}}}} = {\text{k}}\left( {{\text{q}}_{{\text{e}}} - {\text{q}}_{{\text{t}}} } \right)$$

15 $$\frac{{{\text{dq}}_{{\text{t}}} }}{{{\text{dt}}}} = {\text{k}}\left( {{\text{q}}_{{\text{e}}} - {\text{q}}_{{\text{t}}} } \right)^{2}$$

where *k* is rate constant (min^−1^ in Eq. 14 and g·mg^−1^·min^−1^ in Eq. 15), *q*_*e*_ is the concentration of LBG (mg·g^−1^ o.d. fibre) adsorbed at equilibrium, same as in Eq. 10, and *q*_*t*_ is concentration of LBG (mg·g^−1^ o.d. fibre) adsorbed at any time, *t* (min).

Separating variables of the pseudo-first-order model yields:

16 $$\frac{{{\text{dq}}_{{\text{t}}} }}{{{\text{q}}_{{\text{e}}} - {\text{q}}_{{\text{t}}} }} = {\text{kd}}_{{\text{t}}}$$

Using the boundary condition *q*_*t*_ (0 s) = 0 mg·g^−1^ o.d. fibre and integrating, the pseudo-first-order model yields:

17 $$\ln \left( {{\text{q}}_{{\text{e}}} - {\text{q}}_{{\text{t}}} } \right) = { } - {\text{kt}} + {\text{ lnq}}_{{\text{e}}}$$

Separating variables of the pseudo-second-order model yields:

18 $$\frac{{{\text{dq}}_{{\text{t}}} }}{{\left( {{\text{q}}_{{\text{e}}} - {\text{q}}_{{\text{t}}} } \right)^{2} }} = {\text{kd}}_{{\text{t}}}$$

Once again, using the boundary condition *q*_*t*_ (0 s) = 0 mg·g^−1^ o.d. fibre and integrating, the pseudo-second-order model yields:

19 $$\frac{1}{{{\text{q}}_{{\text{e}}} - {\text{q}}_{{\text{t}}} }} - { }\frac{1}{{{\text{q}}_{{\text{e}}} }} = {\text{ kt}}$$

Equation 19 can be rearranged as:

20 $$\frac{{\text{t}}}{{{\text{q}}_{{\text{t}}} }} = \frac{1}{{{\text{k}}\left( {{\text{q}}_{{\text{e}}} } \right)^{2} }} + \frac{{\text{t}}}{{{\text{q}}_{{\text{e}}} }}$$

For pseudo-first-order adsorption kinetics, plotting *ln(q*_*e*_-*q*_*t*_) as a function of *t* will yield a line with slope of  − *k* and intercept of *lnq*_*e*_. For pseudo-second-order adsorption kinetics, plotting $\frac{{t}}{{{q}}_{{t}}}$ as a function of t will yield a line with slope $\frac{1}{{{q}}_{{e}}}$ and intercept of $\frac{1}{{k}{({{q}}_{{e}})}^{2}}$.

### LBG adsorption for strength analysis

An aqueous LBG solution of 0.5 wt% consistency was hydrolyzed at 85 °C for 10 min with constant stirring to produce a transparent viscous solution. The PFI mill was used to refine the pulp to 3000–9000 rev at 10wt% fibre consistency. A NBSK pulp suspension of 1.5 wt% fibre consistency was prepared after refining in the pulp disintegrator for 600 counts, which is equivalent to 1500 rev. The LBG solution was then added to NBSK pulp suspension to the desired dosage with manual stirring for 10 min at 25 °C. Table 2 summarizes the combinations of LBG addition and refining tested. The treated pulp was next diluted to a fibre consistency of 0.3 wt%. A 2 L sample was collected for freeness testing, and the remaining suspension was used for handsheet making. Two to three replicates were conducted for each condition.

**Table 2:** Experimental design for investigation into the effects of LBG dosage and pulp refining on paper strength

Refining (rev)	Dosage relative to o.d. pulp (wt%)
0	0, 0.1, 0.5, 1
3000	0, 0.1, 0.5, 1
6000	0, 0.1, 0.5, 1
9000	0, 0.1, 0.5, 1

### Freeness testing, handsheet preparation and strength analysis

Freeness (Canadian standard method) was tested according to Tappi Method T 227. Handsheets with an average grammage of 60 g·m^−2^ were prepared on a wire of 200 cm^2^ according to Tappi Method T 205. The following handsheet properties were tested: weight and thickness (L&W micrometer), tensile strength (L&W Tensile Strength Tester, Tappi Method T 494), tear index (Elmendorf Tearing Tester, Tappi Method T 414) and burst index (Mullen Tester, Tappi Method T 403). Brightness and scattering coefficient were tested by Technidyne ColorTouch PC according to ISO 2470–1 and TAPPI T 525.

## Results and discussion

### Adsorption kinetics

**Fig. 1:**
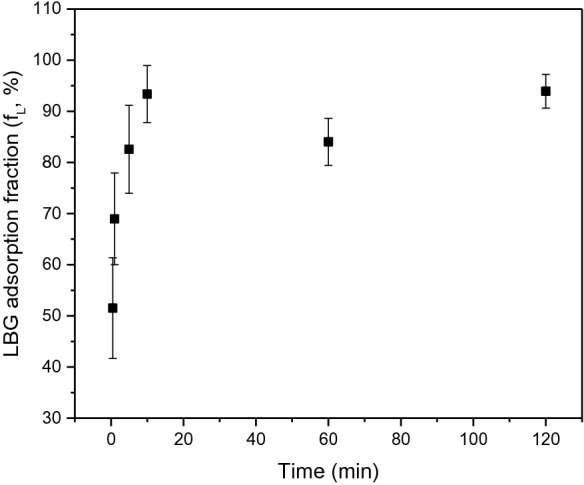
The fraction of LBG adsorbed to NBSK pulp at 25 °C as function of time. LBG dosage was 0.2 wt% relative to o.d. pulp and fibre consistency was 0.5 wt%; all runs were conducted with an agitation rate of 150 r.p.m

The fraction of LBG adsorbed to NBSK pulp (Eq. 1) at 25 °C is plotted as a function of time with LBG dosage of 0.2 wt% relative to o.d. pulp (Fig. 1). The initial adsorption rate was high with more than 52% adsorption in 0.5 min and 82% adsorption within 5 min. After 10 min, the adsorption fraction plateaued at approximately 93%. Adsorption equilibrium was achieved in 10 min as the LBG adsorption fraction was constant from 10 to 120 min. This result is consistent with previous research that found initial adsorption of hemicellulose is rapid and achieves equilibrium in a few minutes (Zakrajšek et al. 2009; Swanson et al. 1949) when low dosages are applied. Adsorption residence time was maintained at 10 min in all subsequent studies.

**Fig. 2:**
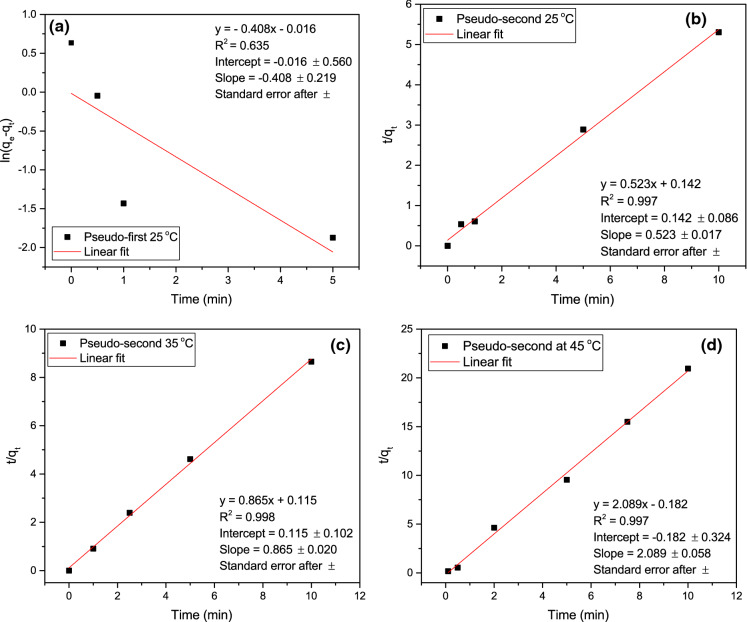
Linear fit of **a** pseudo-first-order kinetics and **b** pseudo-second-order kinetics of LBG adsorption to NBSK pulp at 25 °C. **c** Linear fit of pseudo-second-order kinetics of LBG adsorption to NBSK pulp at 35 °C and **d** at 45 °C

Kinetic plots for LBG adsorption are presented in Fig. 2. The poor fit of the pseudo-first-order model (*R*^*2*^ = 0.635) at 25^ o^C suggests this model cannot describe LBG adsorption kinetics. The pseudo-second-order kinetics model fit well at all tested temperatures (*R*^*2*^ > 0.997). The standard error on slopes are relatively small at all temperatures. Slope is used to calculate *q*_*e*_ (Eq. 10). The standard error on intercepts increase with increasing temperature. The intercept and *q*_*e*_ determined from the slope are used to determine the rate constant. The good fit of the pseudo-second-order model indicates that LBG adsorption is strongly influenced by the concentration of LBG in solution. The second-order reaction may reflect the potential of a single, high molar mass LBG polymer to form multiple bonds. Pseudo-second-order kinetics have also been observed for adsorption of several dyes and chemicals to pulp fibres (Li et al. 2018; Urruzola et al. 2013; Roy et al. 2013; Vučurović et al. 2012).

The adsorption rate constant and equilibrium adsorption amount at 25–45 °C are summarized in Table 3. The rate constant (*k*) at 45 °C is 12 times larger than at 25 °C, and 3.7 times larger than at 35 °C, indicating that LBG adsorption to NBSK pulp is strongly temperature dependent. The adsorption rate in an agitated pulp suspension depends on turbulent transport and Brownian motion (Zakrajšek et al. 2009; van De Ven 1994). When temperature increases, the collision frequency of particle and fibre increases thus increasing adsorption rate with temperature (Table 3). The large confidence intervals associated with the rate constant are due to propagation of the uncertainty of the intercepts predicted in Fig. 2.

**Table 3:** LBG adsorption capacity at equilibrium (*q*_*e*_) and adsorption rate constant (*k*) at temperature range of 25–45 °C as determined by fitting pseudo-second-order kinetics.

Temperature (°C)	Equilibrium adsorption capacity, *q*_*e*_, (mg·g^−1^ o.d. fibre)	Rate constant, *k*, (g·mg^−1^·min^−1^)
25	1.91 ± 0.08	1.93 ± 1.46
35	1.16 ± 0.03	6.51 ± 7.20
45	0.48 ± 0.01	24.03 ± 45.05

95% confidence limits after ± ; all runs were conducted with an agitation rate of 150 r.p.m.

The equilibrium adsorption capacity (*q*_*e*_), however, decreased with increasing temperature from 25 to 45 °C (Table 3). The equilibrium adsorption capacity at 45 °C is 25% of that at 25 °C. Since the amount adsorbed is a result of competition between adsorption and desorption, the decrease of *q*_*e*_ indicates an increase in the escaping capacity of LBG at elevated temperature. To further investigate this temperature effect, adsorption isotherms are discussed below. The activation energy was determined by linear regression of the Arrhenius equation. For LBG adsorption at 25 °C to 45 °C, the activation energy was 99.34 kJ·mol^−1^ (± 9.85 kJ·mol^−1^, 95% confidence interval) with a pre-exponential factor of 4.76 × 10^17^ L·mol^−1^·min^−1^ (Supplementary information Fig. 9). The high activation energy suggests that LBG adsorption to NBSK pulp is a chemisorption process (Laidler 1987). Russo (1959) studied partially methylated LBG adsorption to bleached sulfite pulp and determined the activation energy of adsorption to be 18.4 kJ·mol^−1^ leading Russo (1959) to propose that adsorption is a physical process dominated by diffusion or adsorption via van der Waals forces. The difference between this work and Russo (1959)’s might lie in the agitation. Russo (1959) applied a low agitation rate of 12 r.p.m., while this study applied an agitation rate of 150 r.p.m. When mass transfer limits are high the activation energy will be low reflecting the diffusion process. However, when agitation rate is sufficiently high, the activation energy will reflect the chemical interaction between LBG and cellulose.

### Adsorption isotherms

**Fig. 3:**
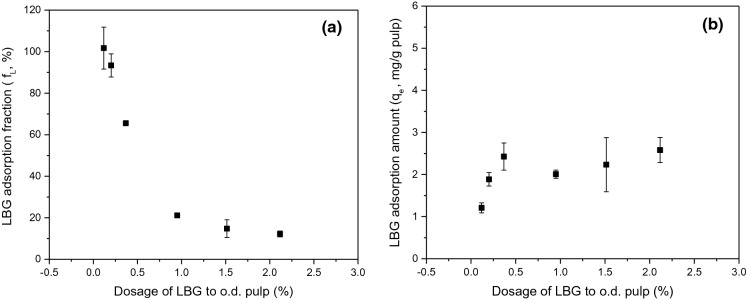
LBG adsorbed to unrefined NBSK pulp fibre as a function of LBG dosage to o.d. fibre after 10 min at 25 °C. **a** The fraction of LBG adsorbed and **b** the equilibrium concentration of LBG adsorbed. Error bars represent standard deviation of 2–4 trials

LBG adsorption isotherms were investigated by varying initial dosage of LBG relative to the weight of o.d. pulp fibre. In Fig. 3a the fraction of LBG adsorbed is plotted as a function of dose while Fig. 3b plots the equilibrium concentration of LBG adsorbed as a function of dose. Increasing dosage causes the fraction of LBG adsorbed to decrease but a greater mass of LBG is retained on the fibre up to a dosage of 0.5 wt%. Given the plateau in mass of LBG adsorbed for dosage between 0.5 and 2.1 wt%, it can be inferred that there is a finite number of adsorption sites on the fibre surface and that LBG adsorption is limited to the fibre surface. This inference is also supported by Wågberg and Hägglund (2001)’s conclusion that polymers with molar mass greater than 48 kDa can only adsorb on the external fibre surface; the weight-average molar mass of LBG was 1215 kDa (± 89 kDa standard deviation) in this work. Hannuksela et al. (2002) also observed that the fraction of guar gum adsorbed decreased with increasing concentration of guar gum; they attributed this to slow diffusion.

**Fig. 4:**
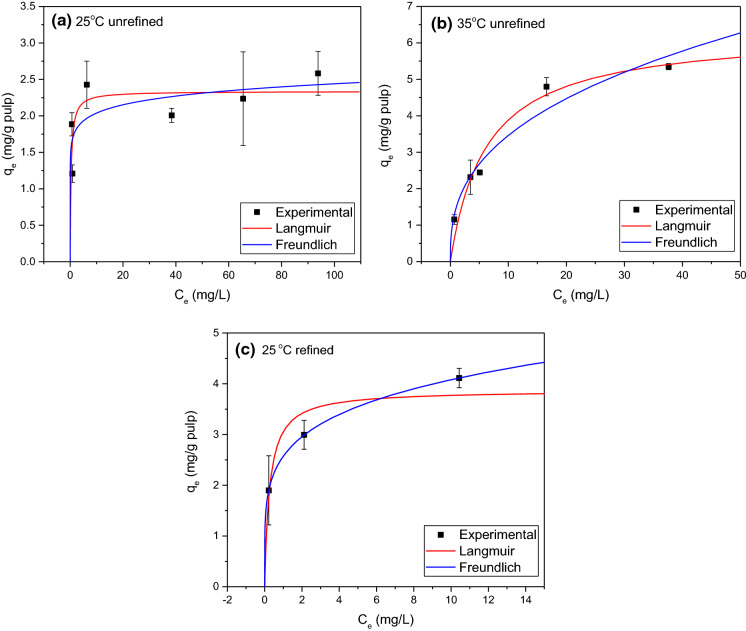
Nonlinear fit of Langmuir model and Freundlich model with experimental results at **a** 25 °C and **b** 35 °C of LBG adsorption to unrefined NBSK pulp, and at **c** 25 °C of LBG adsorption to refined NBSK pulp (3000 rev)

The nonlinear regression results of the Langmuir isotherm model and Freudlich isotherm model at 25 °C and 35 °C with unrefined and refined NBSK pulp are summarized in Table 4. To better illustrate the isotherm fitting, the experimentally determined and predicted adsorption amount, *q*_*e*_, was plotted as a function of aqueous phase LBG concentration at equilibrium (*C*_*e*_) in Fig. 4. Based on Bergmann and Machado (2015), the model with the best fit will have the lowest reduced chi-squared (*χ*^*2*^) and highest ${{R}}_{{adj}}^{2}$. From Table 4, Langmuir isotherm fits better at 25 °C for unrefined NBSK pulp. At 35 °C, both isotherm models fit well with ${{R}}_{{adj}}^{2}$ values close to unity. The temperature effect is discussed in Sect. 3.3.1. For LBG adsorption to refined NBSK pulp (3000 rev) at 25 °C, the Freundlich isotherm model better fit the data as demonstrated by *χ*^*2*^≈0 and ${{R}}_{{adj}}^{2}$ =0.99. This result is discussed in Sect. 3.3.2.

**Table 4:** Summary of nonlinear regression results for Langmuir isotherm and Freundlich isotherm at 25 °C and 35 °C with unrefined and refined NBSK pulp.

Isotherm	Temperature (°C)	Refining (rev)	${{R}}_{{adj}}^{2}$	*Q*_*max*_ (mg·g^−1^)	*K*_*e*_ (L·mg^−1^)	Reduced chi-squared *χ*^*2*^ (mg^2^·g^−2^)
Langmuir	25	0	0.46	2.34 ± 0.20	2.82 ± 1.73	0.13
	35	0	0.95	6.31 ± 0.75	0.16 ± 0.06	0.16
	25	3000	0.73	3.87 ± 1.28	3.75 ± 6.50	0.33

Isotherm	Temperature (°C)	Refining (rev)	${{R}}_{{adj}}^{2}$	*n*	*K*_*f*_ (mg·g^−1^·(mg·L^−1^)^−1/n^)	Reduced chi-squared *χ*^*2*^ (mg^2^·g^−2^)
Freundlich	25	0	0.38	12.82 ± 7.09	1.70 ± 0.25	0.15
	35	0	0.94	2.71 ± 0.50	1.48 ± 0.31	0.19
	25	3000	0.99	5.00 ± 0.04	2.57 ± 0.01	0.00

95% confidence limits after ±

From Table 4 and Fig. 4, it was concluded that LBG adsorption to unrefined NBSK pulp at 25 °C is consistent with Langmuir adsorption principles, indicating that LBG adsorption is a chemisorption process limited to a finite number of sites. According to the Langmuir model, the maximum adsorption capacity (*Q*_*max*_) is 2.34 mg·g^−1^ (± 0.20 mg·g^−1^, 95% confidence interval) at 25 °C. Similar adsorption capacities to pulp fibre (Table 5) were observed for native LBG (1.8–5.0 mg·g^−1^, Gruenhut 1953) and partially methylated LBG (0.61–12.34 mg·g^−1^, Russo 1959). These values are much lower than adsorption capacity for cationic starch (20–66 mg·g^−1^, Table 5). However, the capacity for native corn starch adsorption to pulp was 1.1–4.5 mg·g^−1^ (Cushing and Schuman 1959). Since the charge density of native starch is almost as low as that of LBG, it can be concluded that high positive charge density leads to high adsorption capacity.

**Table 5:** Summary of adsorption capacity and isotherms of polysaccharides adsorption to pulp fibre.

Year	Author	Adsorbate	Adsorbent	Maximum adsorption capacity, *Q*_*max*_ (mg·g^−1^)	Equilibrium rate constant, *K*_*e*_ (L·mg^−1^)	Temperature (°C)	pH	Agitation rate (r.p.m.)	Isotherm model
	This work	Locust bean gum	Northen bleached softwood kraft pulp	2.34 ± 0.20	2.82 ± 1.73	25	5.2–5.8	150	Langmuir
2009	Zakrajšek et al	Cationically modified starch	Hardwood sulphate short fibres	66	40	25	7.5 ± 0.2	500	Modified Langmuir
2003	Shirazi et al	Cationic starch	Unbleached black spruce TMP	20	N.A	N.A	5.2	200	Langmuir
1959	Cushing and Schuman	Native corn starch	Bleached sulphite pulp	1.1–4.5*	N.A	91–96	5.0–5.1	N.A	N.A
1959	Russo	Partially methylated LBG	Bleached sulfite pulp	0.61–12.34*	N.A	25	6.5	12	N.A
1953	Gruenhut	Locust bean gum	Rosin sized kraft pulp	1.8–5.0*	N.A	22	N.A	N.A	N.A

95% confidence limits after ±

*No isotherm fitting, therefore, adsorption amount range is indicated

The equilibrium constant (*K*_*e*_) calculated from the Langmuir model is approximately 2.82 L·mg^−1^ (± 1.73 L·mg^−1^, 95% confidence interval) at 25 °C. The large confidence interval reflects the limited number of data points used to prepare the model. The equilibrium constant for cationic starch adsorption to pulp fibre is much higher, for example Zakrajšek et al. (2009) reported *K*_*e*_ = 40 L·mg^−1^. This high equilibrium constant could be due to the high concentration of adsorbed cationic starch resulting from the natural attraction between negatively charged pulp fibres and cationic starch. Unlike cationic starch, LBG is a natural carbohydrate polymer with a negative surface charge (de Jong and van de Velde 2007). The repulsive forces between LBG and NBSK pulp fibre will reduce adsorption capacity. Consequently, LBG adsorption to unrefined NBSK pulp is characterized by low adsorption capacity and a relatively low equilibrium constant.

## Factors influencing adsorption

### Effect of temperature

**Fig. 5:**
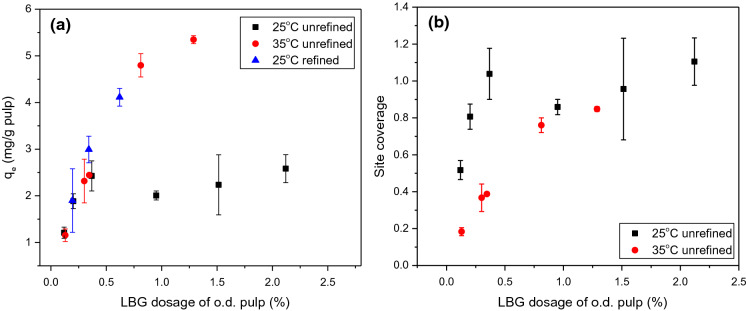
**a** Amount of LBG (mg·g^−1^ o.d. pulp) adsorbed to NBSK pulp after 10 min as a function of LBG concentration in aqueous solution (mg·L^−1^) at 25 °C, 35 °C for unrefined and refined NBSK pulp. **b** Fibre surface site coverage (*θ*) calculated from Langmuir model at 25 °C and 35 °C for unrefined NBSK pulp

The influence of temperature on LBG adsorption to NBSK pulp fibre at two temperatures, 25 °C and 35 °C on o.d. fibre, is reported in Fig. 5a. At low dosage levels (< 0.5wt%), LBG adsorption amount is comparable at both temperatures; the adsorption amount to refined pulp at 25 °C is similar to the unrefined materials. At low dosage, it appears that LBG affinity to NBSK pulp fibre is not affected by temperature. At high LBG dosage levels, the amount of LBG adsorbed to fibre at 35 °C is much higher than that at 25 °C. The amount of LBG adsorbed to refined pulp at 25 °C is comparable to the amount adsorbed to unrefined pulp at 35 °C. When LBG concentration in solution increased (> 0.5wt%), the adsorption amount differentiates depending on temperature and surface condition of NBSK pulp.

From Table 4 and Fig. 4, it was found that adsorption at 25 °C is best described by the Langmuir model while adsorption at 35 °C can be described by either the Langmuir model or Freundlich model. From the Langmuir model, the maximum adsorption capacity (*Q*_*max*_) of pulp fibres at 35 °C was calculated to be 6.31 mg·g^−1^ (± 0.75 mg·g^−1^, 95% confidence interval) o.d. pulp. This is greater than the *Q*_*max*_ determined at 25 °C, 2.34 mg·g^−1^, suggesting that more surface sites are accessible at higher temperature. However, increasing temperature could hardly increase the total surface area of pulp fibres. Given the good fit of both models, neither can be definitively selected. Instead, we suggest that an alternate mechanism may be involved. There may be multi-layer adsorption at 35 °C, and the adsorption occurs layer-by-layer (a pseudo-Langmuir mechanism). By this reasoning, the number of sites in each adsorption layer is constant and the increase in predicted *Q*_*max*_ at 35 °C reflects the presence of multiple layers.

Adsorption–desorption equilibrium is a dynamic process. Surface site coverage will vary with adsorption conditions. The Langmuir equilibrium constant (*K*_*e*_) was determined to be 0.16 L·mg^−1^ (± 0.06 L·mg^−1^, 95% confidence interval) at 35 °C, which is lower than *K*_*e*_ = 2.82 L·mg^−1^ at 25 °C. This decrease could be caused by lower concentration of adsorbed LBG ([*C*_*e*_*S*_*e*_], Eq. 8) and/or increased surface site concentration ([*S*_*e*_], Eq. 8). From Fig. 5a, the equilibrium adsorption amount (*q*_*e*_) increased with increasing temperature at higher dosage levels, indicating that the adsorbed LBG concentration (*C*_*e*_*S*_*e*_) increases with increasing temperature. Thus, the lowered *K*_*e*_ value at 35 °C could due to the increased surface site concentration.

Site coverage on unrefined NBSK pulp increased rapidly at LBG dosages less than 0.2wt% at 25 °C (Fig. 5b). However, the dependence on dosage diminishes as full coverage is approached. At 35 °C, the reduced dependence on dosage could result from increased maximum adsorption capacity (*Q*_*max*_). Past studies have shown paper tensile strength improvement is not proportional to hemicellulose dosage, and the increase in paper tensile strength diminishes as dosage is increased. Hannuksela et al. (2004) reported GGM dosage of 0.8 wt% o.d. fibre increased tensile strength by 13% but a higher dosage of 1.6 wt% o.d. fibre caused only a small additional increase in tensile strength. The most significant improvement in tensile strength was achieved at GGM dosage less than 0.1 wt% dried fibre (Hannuksela et al. 2004). Our results help explain Hannuksela et al. (2004)’s observations. When LBG dosage was approximately 0.12 wt%, the coverage of fibre sites at 25 °C was 0.52 (Fig. 5b). Full coverage was obtained when the dosage was increased to 2.12 wt% of o.d. pulp fibre. As the fibre surface becomes saturated, it is likely that improvement in inter-fibre bonding will be limited leading to small gains in paper tensile strength, even after addition of excess LBG.

#### Effect of refining

Table 4 and Fig. 4c clearly demonstrate that the adsorption to lightly refined pulp at 25 °C is best described by the Freundlich model. Adsorption to unrefined pulp at 25 °C was best described by the Langmuir model. The Freundlich model is used to describe multi-layer adsorption on a non-uniform and heterogeneous surface. The calculated adsorption constant (*n*) is 5.00 (± 0.04, 95% confidence interval), and *K*_*f*_ the equilibrium constant is 2.57 mg·g^−1^·(mg·L^−1^)^−1/n^ (± 0.01 mg·g^−1^·(mg·L^−1^)^−1/n^, 95% confidence interval). This change in adsorption isotherm relative to the unrefined NBSK pulp is likely a result of the heterogeneity of the fibre surface created by fibrillation during refining.

#### Effect of salt addition

**Fig. 6:**
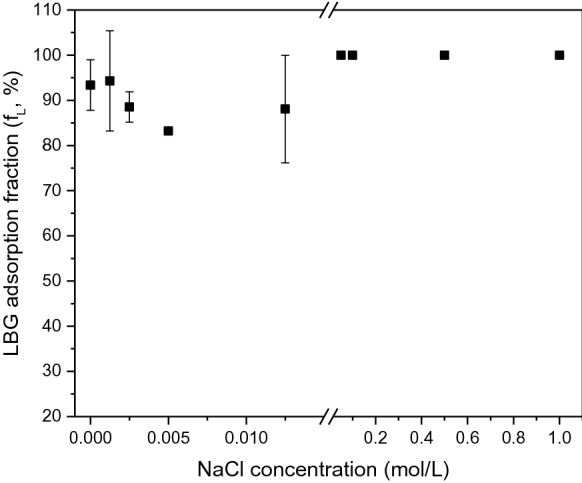
The adsorbed fraction of LBG on NBSK pulp fibre with varying sodium chloride concentration 0–1.0 mol·L^−1^ after 10 min at 25 °C, LBG dosage 0.2 wt% of o.d. fibre

LBG adsorption to NBSK pulp fibre in response to sodium chloride addition was investigated at 25 °C for 10 min with LBG dosage 0.2 wt% relative to o.d. fibre; sodium chloride concentration was varied from 0–1 mol·L^−1^ (Fig. 6). The LBG adsorption fraction was approximately 85–100% and independent of sodium chloride concentration at concentrations from 0–1.0 mol·L^−1^ (Fig. 6). The small effect of sodium chloride might due to the small electrostatic repulsion between LBG and fibre and the low negative charge density on LBG (de Jong and van de Velde 2007). This trend is consistent with Hannuksela et al. (2002)’s research that guar gum and GGM adsorption were unaffected by addition of less than 0.1 mol·L^−1^ sodium chloride.

#### Effect of pH

**Fig. 7:**
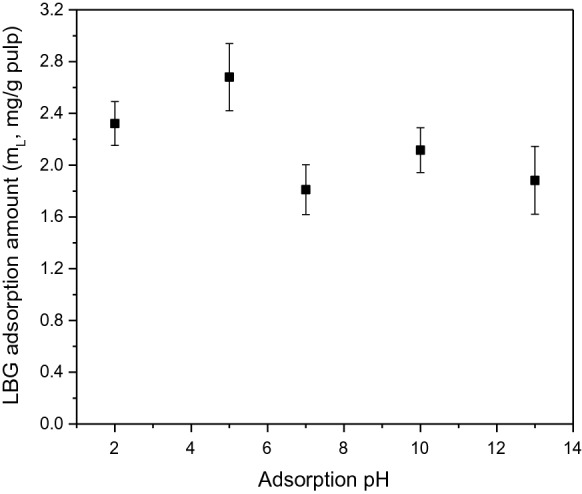
The adsorbed amount of LBG on NBSK pulp fibre with varying pH of pulp suspension after 10 min at 25 °C, LBG dosage 0.2 wt% of o.d. fibre

LBG adsorption to NBSK pulp was investigated after 10 min at 25 °C with LBG dosage 0.2 wt% of o.d. fibre (Fig. 7) while buffer pH varied from 2 to 13. The amount of LBG adsorbed ranged from 1.8–2.7 mg·g^−1^ of o.d. fibre with maximum standard deviation of 0.26 mg·g^−1^ of o.d. fibre (Fig. 7), indicating adsorption was not strongly affected by pH. Adsorption was slightly higher at pH 2 and 5 due to the undissociated hydroxyl and carboxyl groups on LBG and reduced repulsive forces. At high pH, hydroxyl and carboxyl groups deprotonate more easily increasing repulsive forces between fibre and LBG thus lowering adsorption. However, the differences are small due to LBG’s low negative charge density on LBG. Hannuksela et al. (2002) reported that pH (5 and 8) had no influence on guar gum adsorption to softwood kraft pulp fibre. This observation is probably due to guar gum’s weak negative charge and the limited range of pH tested.

### Paper strength enhancement by LBG adsorption

**Fig. 8:**
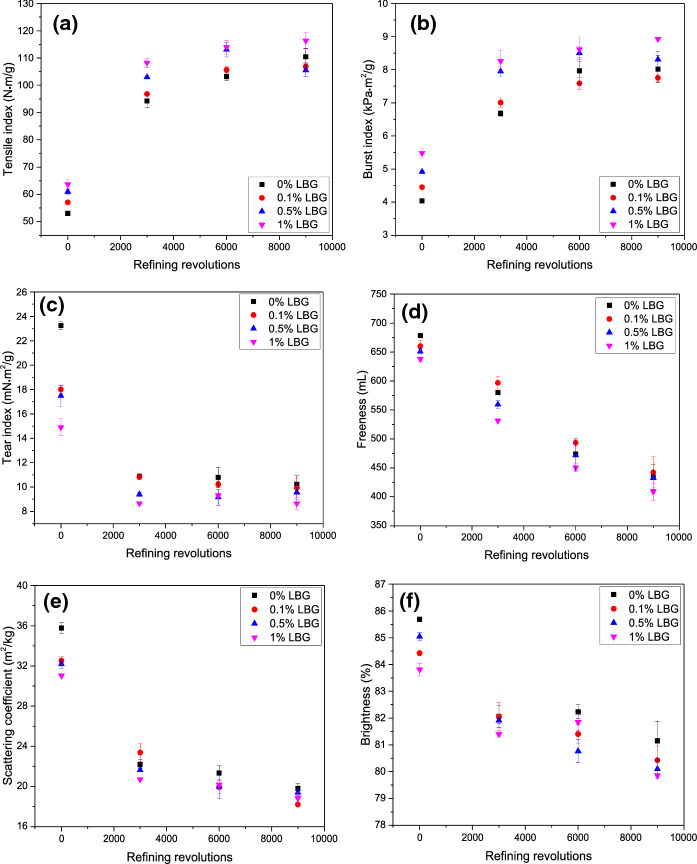
NBSK pulp and paper properties as a function of LBG dosage and PFI refining level: **a** tensile index, **b** burst index, **c** tear index, **d** freeness, **e** scattering coefficient and **f** brightness. LBG adsorption was conducted at 25 °C for 10 min

Pulp and paper properties including tensile, burst, tear index, scattering coefficient, brightness, and pulp freeness were plotted as a function of PFI refining revolutions and LBG dosage in Fig. 8. Adsorption was conducted after refining. Refining and dosage positively influenced tensile strength and burst strength. Tear strength, freeness, scattering coefficient, and brightness, in contrast, decreased with increasing refining or dosage level.

It is well-established that refining increases NBSK paper strength (Fig. 8). Tensile index and burst index increased with refining level until 6000 rev and then plateaued. Refining to 9000 rev without LBG adsorption doubled the tensile index of unrefined NBSK paper. However, as highlighted by Leech (1954), paper strength does not increase continuously with bonding strength as at higher levels of bonding, handsheet strength becomes more dependent on individual fibre strength. Leech (1954) concluded that tensile and burst strength of bleached sulfite pulp plateau after refining to approximately 6000 rev, which is consistent with the results in Fig. 8a, b.

Mechanical refining had a much greater effect on tensile strength than LBG adsorption. Miletzky et al. (2015) similarly reported that the effects of hemicellulose addition are minimal if high refining is applied. In this work, the maximum increase in tensile index due to adsorption of LBG to unrefined pulp was 20.1%. In comparison, tensile index after refining was approximately double that of unrefined pulp. However, LBG adsorption can reduce the degree of refining and energy required to reach a target tensile index. For example, pulp must be refined to 2000 rev to reach a tensile index of 80 N·m·g^−1^ without LBG adsorption but only 1000 rev were required to achieve the same index if the pulp is treated with 1wt% LBG. Swanson (1950) likewise reported that refining time was reduced by approximately 70% with addition of 0.5 wt% LBG.

Burst strength demonstrated a similar trend as tensile strength (Fig. 8b). The maximum burst index also doubled after applying 1 wt% LBG to highly refined pulp (9000 rev). Enhanced tensile index and burst index could be due to either increased bonding area or an increased number of bonds and bond strength (Leech 1954). Similar trends were reported by Swanson (1950).

As expected, tear index decreased with refining and LBG addition possibly due to stronger bonding between fibres (Fig. 8c). At 9000 rev, tear index decreased 62.8% after adsorption with 1 wt% LBG. A drop in tear index normally accompanies increased tensile strength (Hannuksela et al. 2004; Leech 1954; Swanson 1950). Tearing occurs due to the breakage of individual fibres or individual fibres being pulled from the sheet matrix. At higher bonding levels, individual fibres will break and this requires less energy than removing fibres from the sheet matrix (Hannuksela et al. 2004; Leech 1954; Swanson 1950).

Freeness is a measurement of pulp drainage (Smook and Kocurek 1982) and reflects fines content, flexibility and the degree of external fibrillation (Niskanen 1998). With increasing refining energy and LBG adsorption, freeness decreases (Niskanen 1998). With the application of 1 wt% LBG to NBSK pulp refined at 3000 rev a tensile strength of 108 N·m·g^−1^ could be achieved at a freeness of 531 mL (Fig. 8d). To achieve a similar tensile strength solely by refining, required 9000 rev and resulted in the lower freeness of 434 mL. Thus, LBG adsorption resulted in better drainage properties at equal tensile strength with less refining energy.

Scattering coefficient decreased with refining level and LBG dosage (Fig. 8e), which suggests more bonding occurs after LBG adsorption and refining. The greatest change in scattering coefficient on LBG adsorption, a 14% decrease, occurs between unrefined pulp without LBG and unrefined pulp with 1wt% LBG. Increasing refining level diminished the impact of LBG dosage on scattering coefficient (Fig. 8e), indicating that refining is the dominant factor for bonding. At 6000 rev and 9000 rev, the scattering coefficients are independent of LBG dosage, thus indicating there is a finite degree to which bonding can be improved. The overall trends of scattering coefficient agree with the trend in tensile strength with respect to refining and LBG adsorption. Consequently, NBSK paper tensile strength enhancement is mainly due to bonding formation.

Brightness decreased with increased refining and LBG dosage level (Fig. 8f). However, as the chromophores in the pulp are not changed and the LBG is colourless, the main reason for the decrease in brightness must be the reduction in the scattering coefficient arising from increased bonded area (Ek et al. 2009; Parsons 1941; Simmonds and Coens 1967).

## Conclusion

Locust bean gum adsorption and its performance as a strength additive was studied. The adsorption rate followed pseudo-second-order chemisorption kinetics. The adsorption rate constant increased rapidly with temperature from 25 to 45 °C, but the amount adsorbed at equilibrium decreased.

Langmuir and Freundlich models were used to describe LBG adsorption with equal fit and both were used to gain physical insight. The maximum LBG adsorption capacity of NBSK pulp fibre was comparable to that of native starch to pulp. The mechanism of LBG adsorption to cellulose fibres is complex and may involve multi-layer adsorption to a finite number of sites. Refining to 3000 rev increased surface heterogeneity of NBSK pulp as evidenced by the excellent fit of the Freundlich model.

Increasing temperature from 25 to 35 °C caused LBG adsorption to increase at dosage level higher than 0.5wt%. Sodium chloride addition (0–1.0 mol·L^−1^) had little effect on adsorption and adsorption increased slightly at pH 2–5. Both observations are likely due to the low negative charge density on LBG.

Refining and LBG dosage increased NBSK paper tensile strength and burst strength. Tensile and burst strength plateaued when refining over 6000 rev, and strength gains were small for LBG dosage greater than 0.5 wt%. However, addition of LBG enabled a reduction in refining revolutions to achieve a target tensile strength with higher freeness compared to solely refining. Tear index, brightness and scattering coefficient decreased, likely due to greater inter-fibre bonding.

## Supplementary Information

Below is the link to the electronic supplementary material.

Supplementary file1 (DOCX 64 kb)
