# Supplementary material for: Locust bean gum adsorption onto softwood kraft pulp fibres: isotherms, kinetics and paper strength
Source: Cellulose (Lond). 2021 Oct 1;28(16):10183–201. doi: 10.1007/s10570-021-04133-w (PMC8570309; doi:10.1007/s10570-021-04133-w)
Supplement: Supplementary file 1 — Supplementary file1 (DOCX 64 kb) [file 10570_2021_4133_MOESM1_ESM.docx]

Supplementary Information

A1. Activation energy determination

**Fig. 9** Linear regression of Arrhenius equation to determine activation energy of LBG adsorption to NBSK pulp at 25 ^o^C to 45 ^o^C based on the pseudo-second order kinetic model.

**Table 6** Compositional analysis of NBSK pulp fibres. Weight fraction (%) of components is presented on a moisture free (o.d. fibre) basis; error limits are standard deviation calculated from 2 replicates.

| Composition (%) | NBSK pulp |
| --- | --- |
| Alpha cellulose | 83.66 ± 0.20 |
| Total carbohydrates | 17.48 ± 0.08 |
| Arabinan | 0.71 ± 0.01 |
| Galactan | 0.26 ± 0.00 |
| Glucan | 1.63 ± 0.01 |
| Xylose | 7.81 ± 0.00 |
| Mannan | 5.12 ± 0.05 |
| ASL | 1.46 ± 0.02 |
| Total mass balance | 102.6 ± 0.3 |
